# Study on Incompatibility of Traditional Chinese Medicine: Evidence from Formula Network, Chemical Space, and Metabolism Room

**DOI:** 10.1155/2013/352145

**Published:** 2013-11-24

**Authors:** Wei Long, Xiao-Dong Zhang, Hong-Ying Wu, Jin Jin, Guang-Yun Yu, Xin He, Hao Wang, Xiu Shen, Ze-Wei Zhou, Pei-Xun Liu, Sai-Jun Fan

**Affiliations:** Tianjin Key Laboratory of Radiation Medicine and Molecular Nuclear Medicine, Institute of Radiation Medicine, Chinese Academy of Medical Sciences and Peking Union Medical College, Tianjin 300192, China

## Abstract

A traditional Chinese medicine (TCM) formula network including 362 TCM formulas was built by using complex network methodologies. The properties of this network were analyzed including network diameter, average distance, clustering coefficient, and average degree. Meanwhile, we built a TCM chemical space and a TCM metabolism room under the theory of chemical space. The properties of chemical space and metabolism room were calculated and analyzed. The properties of the medicine pairs in “eighteen antagonisms and nineteen mutual inhibitors,” an ancient rule for TCM incompatibility, were studied based on the TCM formula network, chemical space, and metabolism room. The results showed that the properties of these incompatible medicine pairs are different from those of the other TCM based on the analysis of the TCM formula network, chemical space, and metabolism room. The lines of evidence derived from our work demonstrated that the ancient rule of TCM incompatibility, “eighteen antagonisms and nineteen mutual inhibitors,” is probably scientifically based.

## 1. Introduction

Chinese formula is the most common use form of TCM in clinical treatment. The theory of TCM incompatibility is the fundamental principle that should be followed for correct clinical medication, which has a direct relationship with effectiveness and safety of TCM formulas. With the accumulation of practice and experience for thousands of years, the theoretical system of TCM and formulas was gradually taking form and passed it down, such as “Harmony in Four Properties and Five Flavors,” “Sovereign, Minister, Assistant and Guide in Formulas”, and “eighteen antagonisms and nineteen mutual inhibitors,” a special rule for TCM incompatibility in formulas. Since the beginning of the 21st century, the modernization of TCM in China has remarkably proceeded. However, up to present, interpreting ancient theory of TCM in modern scientific language is still a challenging problem for us to make clear the traditional subjects, like the incompatibility of TCM.

The complexity characteristic of traditional Chinese medicine has been generally accepted for many years [1]. The theory of complicated system provides a new cutting point for the present TCM researches. The characteristics of TCM, like complexity and systematicness, make the methodologies of complex system theory adapted naturally to the investigations on TCM. The complex network method is frequently used in studying the complex systems in recent years [2, 3], particularly in biological researches. Thanks to the complex network technologies, to some extent, Systems Biology has been developed from academic theory to practical application [4–7]. In recent years, systems Biology has been introduced into the TCM researches. Li et al. carried out a series of studies centering on TCM Syndrome theory and Chinese herbal property with complex network technology in Systems Biology and made their own achievements [8–13].

On the other hand, the theory of chemical space has drawn widespread attention as soon as it was proposed. Theoretically, chemicals can be characterized by a wide range of “descriptors,” such as their molecular mass, lipophilicity, and topological features. “Chemical space” is a term often used in place of “multi-dimensional descriptor space”: it is a region defined by a particular choice of descriptors and the limits placed on them. In the context of this insight, chemical space is defined as the total descriptor space that encompasses all the small carbon-based molecules that could in principle be created [14]. It describes things at the standpoint of macrochemical perspective and tends towards conclusions on the integral view. It has been applied preliminarily in TCM studies [15]. Based on the theory of chemical space, we created TCM chemical space and TCM metabolism room in this work by using TCM chemical and metabolic properties.

Up to now, our understanding of the whole law of TCM formula is still poor, due to the complexity of the traditional theories, such as incompatibility of TCM, and less documents available in this field. In order to resolve the mystery of the ancient rule of TCM incompatibility, “eighteen antagonisms and nineteen mutual inhibitors,” scientific experiments based on chemistry and pharmacology were carried out recently [16, 17]. In this study, based on our preliminary work [18], we constructed TCM formula network, chemical space, and metabolism room by collecting massive data with complex network and chemical space methodologies. Property analysis was preformed based on the formula network, chemical space, and metabolism room to get the knowledge of TCM formulas, and efforts were made to interpret the ancient rule of TCM incompatibility, “eighteen antagonisms and nineteen mutual inhibitors,” from a new viewpoint. 

## 2. Materials and Methods

### 2.1. TCM Formula Network

#### 2.1.1. Data Collection

Three hundred and sixty-two TCM formulas were collected into our formula database in the light of *Chinese Medical Formulas* [19], the Textbook for TCM Colleges and Universities edited by the National Educational Ministry. These formulas are all classic and effective and confirmed by numerous clinical studies, with authority and reliability both ensured. 

#### 2.1.2. Network Construction

The TCM formula network was constructed with all the 330 Chinese drugs collected in the formula database. The Chinese drugs were defined as the network nodes, and the relationships among these drugs were defined as the network edges. It was stipulated that there was a special relationship between the two Chinese drugs if they appeared in the same formula; then we give them a network edge by connecting the two drugs or two nodes. Consequently, these network nodes and edges make up the whole TCM formula network. Excel combining with programming script was used to process the original data, and then they were converted to Pajek file format by Creatpajek program [20].

#### 2.1.3. Network Analysis

The files obtained above were imported into network analysis software, such as Pajek [21] and ORA [22, 23]. The frame chart was subsequently drawn, and topology analysis was performed as follows [24].

(1) The distance (*d*_*ij*_) between the nodes *i* and *j* in the network is defined as the number of edges on the shortest path that connected the two nodes. The maximum distance between any of two nodes, which is noted as *D*, is defined as the diameter in the network. The average path length *L* in the network is defined as the average distance of any two nodes.
(2) Assuming a node (*i*) has *K*_*i*_ sides to connect it with other nodes, then these *k*_*i*_ nodes are called the neighbors of *i*. Obviously there are at most *k*_*i*_ (*k*_*i*_ − 1)/2 sides among *k*_*i*_ nodes. The ratio of the number of sides that actually exist to that which might exist in the overall is defined as clustering coefficient of the node *C*_*i*_.
(3) In the properties of individual node, degree is a simple and important concept. The degree of node, *k*_*i*_, is defined as the number of the other nodes connecting to this node (*i*). The average of all the degrees is called the average degree of the network. The distribution of degree in the network can be described with a distribution function *P*(*k*) which means the probability of a randomly selected degree *k*. Numerous studies indicate that the distribution of degree can be described in the form of power law:
  (1) $$\begin{array}{c} P\left(k\right)\propto k^{-\gamma }. \end{array}$$

The network that is featured by power-law distribution is also called scale-free distribution network.

### 2.2. TCM Chemical Space

**Figure 1:**
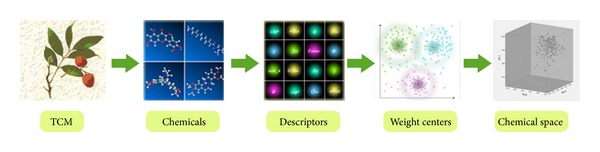
The workflow of constructing TCM chemical space.

The construction of TCM chemical space was based on the database of Chinese Medicine Chemistry which was developed in our previous work [25], and 383 commonly used TCM are included in this database. The chemical components of every TCM, 8514 compounds in total, are collected in the database. TCM chemical space was constructed according to the following steps shown in Figure 1.

#### 2.2.1. Calculation of Descriptors

The descriptor calculation modules of software CODESSA [26] and MOE [27] were used to calculate the descriptors of all chemical constituents of each TCM in the database. There are 120 descriptors involved in all, including structural descriptors, fragmental descriptors, electrical descriptors, topology descriptors, and space descriptors. These descriptors comprehensively describe the physic-chemical properties of the chemical constituents of each TCM in various perspectives and levels, which paved the way for the construction of TCM chemical space.

#### 2.2.2. Weight Center Treatment

**Figure 2:**
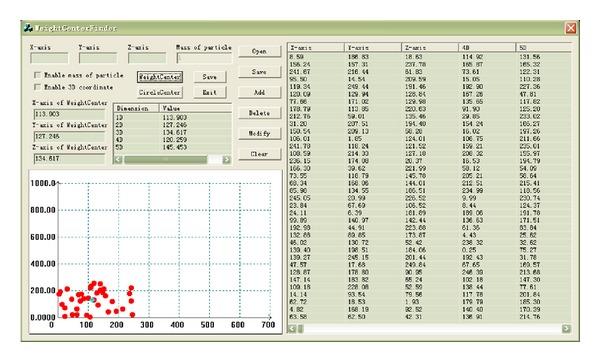
Graphical user interface of software “WeightCenterFinder.”

Each of 120 descriptors was defined as a dimensionality. Then all the chemical constituents of each TCM were distributed in a multidimensional chemical space. According to the theory of Chinese medicine chemistry, the property of each TCM is mostly determined by its chemical constituents. However, the number and type of chemical constituents in each TCM vary from tens to hundreds. It is hard to describe the properties of TCM by using such a wide variety of chemical constituents in a chemical space. After instant searches and creations, we developed a new method called weight center treatment that is employed to gather all the constituents of every TCM to a point, which bring together the chemical properties of all the constituents of a TCM and dexterously balance the differences among the chemical constituents. As a result, the whole property of a TCM can be described in a chemical space from a macro perspective. It is assumed that all the particles have the same weight in this work. Each TCM can be presented as a separate unit in the chemical space. Thereby, all the TCM can be redefined chemically by this method of chemical space. The detail of this treatment was described in reference [26]. The weight center treatment is accomplished by the software of “WeightCenterFinder” (Figure 2), which is compiled on Visual C++ 6.0 platform.

#### 2.2.3. Dimension Reduction in Chemical Space

It is difficult to describe the chemical properties of the objects in multidimensional chemical space. In the present study, in order to reduce the chemical dimensions, we employed the method of principal component analysis (PCA) to obtain the eigenvalues and eigenvectors by calculating relevant matrices and diagonalization of the 120 descriptions. Matrix of eigenvectors is used as a transformational matrix to bring the largest percentage of information into the new units of relevant matrix. It can be plotted when the number of units reduced to three. Thereby, the data of multidimensional descriptors can be effectively mapped to 3D diagram. If the cumulative contribution rate of the three main units reaches 80%, it means that the three main units included message of all descriptors basically. The PCA and other statistical analyses were all performed by using SPSS 16.0 [28].

#### 2.2.4. Calculation of Chemical Spatial Distance

The chemical spatial distance can display the relationship of affinity between the two TCM, and the formula for calculating chemical spatial distance is given herein: *D* = [(*X*_*j*_ − *X*_*i*_)^2^ + (*Y*_*j*_ − *Y*_*i*_)^2^ + (*Z*_*j*_ − *Z*_*i*_)^2^]^1/2^.

Here (*X*_*i*_, *Y*_*i*_, *Z*_*i*_), (*X*_*j*_, *Y*_*j*_, *Z*_*j*_) represent the coordinates of any two dots in chemical space.

**Figure 3:**
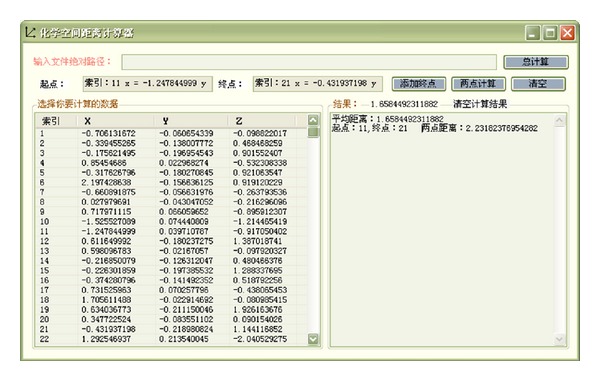
Graphic user interface of chemical spatial distance calculator.

The calculation of chemical spatial distance is accomplished by self-developed chemical spatial distance calculator (Figure 3). Affinity and compatible relationship among TCM is analyzed by calculating chemical spatial distance.

### 2.3. TCM Metabolism Room

In this study, we made a metabolism room involved with P450 enzymes, which are the most important factors that influence the incompatibility property of TCM. Through analysis of this metabolism room, we tried to find evidence for interrupting the rule of “eighteen antagonisms and nineteen mutual inhibitors.” All the TCM chemicals were derived from the database of Chinese Medicine Chemistry, with 383 commonly used TCM and 8514 TCM chemicals involved. TCM metabolism room was built by a similar workflow like the TCM chemical space, under the basic theory of chemical space. In the construction process, there were four steps as follows. 

#### 2.3.1. Molecular Docking

Fifteen P450 enzymes, the most critical ones related to drug and TCM metabolism, were selected as targets for TCM chemicals in molecular docking. The crystal structures of these enzymes were collected from PDB, with the names and their PDB code as follows: CYP 1A1 (PDB code: 4I8V), CYP 1A2 (PDB code: 2HI4), CYP 1B1 (PDB code: 3PM0), CYP 2A6 (PDB code: 1Z10), CYP 2A13 (PDB code: 2PB5), CYP 2B4 (PDB code: 3TMZ), CYP 2B6 (PDB code: 3UA5), CYP 2C5 (PDB code: 1NR6), CYP 2C8 (PDB code: 2VN0), CYP 2C9 (PDB code: 1R9O), CYP 2C18 (PDB code: 2H6P), CYP 2C19 (PDB code: 4GQS), CYP 2D6 (PDB code: 3TDA), CYP 2E1 (PDB code: 3T3Z), and CYP 3A4 (PDB code: 3NXU). Molecular docking was implemented by Glide, a docking program in Schrodinger software package. Protein preparation, chemical disposure, and other operations were all carried out in Maestro, the molecular modeling environment of Schrodinger software. The docking score of each TCM chemical towards each P450 enzyme will be assigned as a metabolic value for this chemical. Then, each TCM chemical will have 15 metabolic values corresponding to the 15 P450 enzymes. These metabolic values describe the whole P450 metabolic properties of this chemical from an overall perspective. 

#### 2.3.2. Weight Center Treatment

The same treatment used in TCM chemical space was applied in this process. As the treatment result, each TCM will be presented by a weight center, which was concentrated from all the chemicals in this TCM, in a multiple dimensional metabolism room. 

#### 2.3.3. Dimension Reduction

The same treatment used in TCM chemical space was applied in this process. The original 15 dimensions were reduced into 3 dimensions by using PCA, with over 80% of the cumulative contribution rate from these 3 main units.

#### 2.3.4. Calculation of Metabolism Room Distance

We applied the same method in TCM chemical space to calculate the metabolism room distance.

## 3. Results and Discussion

### 3.1. TCM Formula Network

**Figure 4:**
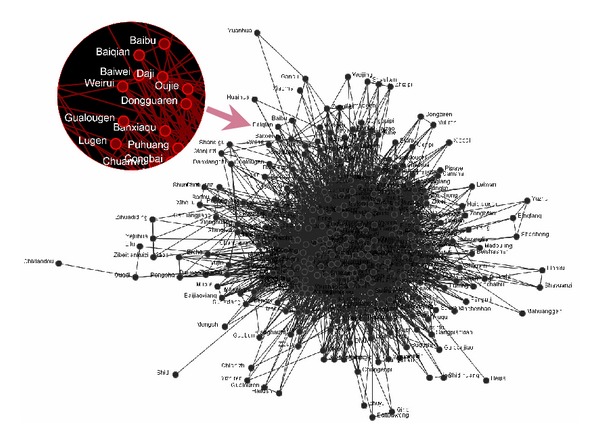
The map of TCM formula network (a partial enlarged illustration on the upper left).

The TCM formula network we constructed has 330 nodes and 5236 edges in all. The network structure chart is drawn with the software ORA. We can see the results in Figure 4.

The topological parameters of the network were calculated by Pajek; for main parameters, see Table 1.

There are 330 nodes in this network, and the number of edges reached 5258, with 31.7 as the average degree of each node, which indicate a high network density and considerably close connection among TCM. Most of TCM are likely to have good comparability with each other, which is in line with the actual application of TCM. The diameter of the network is 5, and the average path length is 2.17, which indicate that the TCM formula network is featured as a small-world network. Generally, the clustering coefficient of the network ranges from 0 to 1. The bigger the coefficient, the higher the clustering property. The clustering coefficient of the TCM formulas network is 0.726, which indicates a high clustering property of this network.

**Figure 5:**
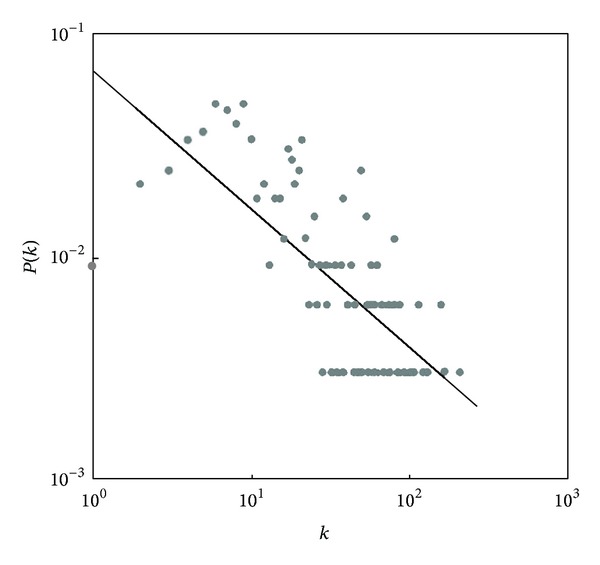
The degree distribution chart of TCM formulas network.

As we know, there are hundreds of medicines frequently used in the TCM formulas, and the properties of TCM are different from each other, such as “Four Properties and Five Flavors,” “Ascending, Descending, floating and sinking.” However, the different TCM in the same formula can be used as a whole to cure the same kind of disease. This may be because the TCM of different properties in the same formula interacts with each other, and these interactions make them tended to be homogenous and compatible. Turning to the TCM formula network, the high clustering property of the network probably gives us a clue to explain it. The analysis results of degree distribution showed that the TCM formulas network is featured by partly scale-free property (Figure 5). The degree distribution function is described as follows:

(2) $$\begin{array}{c} P\left(k\right)\propto k^{-0.6237},\;\;\gamma =0.6237. \end{array}$$

According to the theory of complex networks, the higher the value of power exponent, the less homogenous the network. Thus, a low value of power exponent indicates that the hubs and the key nodes play vital roles in the network. The *γ* of this network is 0.6237, which is smaller than the general scale-free network, which shows that the key nodes in TCM formula network are in great numbers and play critical roles in this network. It can be concluded that the commonly used TCM occupy an important position in forming of the formulas. Additionally, the scale-free network is usually of robustness, which is also called antistrike capability. Interestingly, the clinical use of TCM formulas exhibits the same characteristic as well. As is known to us, some Chinese medicines have substitutes in clinical usage; for instance, rhinoceros horn can be substituted by buffalo horn. It is normal practice for traditional Chinese physicians to substitute, add, and reduce some medicine in the same formula according to actual needs. But it will not change the main property and function of this formula in clinic. A traditional Chinese doctor can still make a prescription by using the principle of substitution in the case of some medicine lacking. On the other hand, they are usually making different prescriptions to the same disease case because of different habits of prescribing, but they can still achieve the same therapeutic purposes. Probably, the robustness property of the TCM formulas network can give us insight on explaining all the phenomena mentioned above.

Network path analysis of the TCM pairs derived from “Eighteen Antagonisms and Nineteen Mutual Inhibitors” was performed based on the formula network (seen in Figure 8). The path distance of the network nodes represents their affinity relationship. The result shows that the average path distance between the TCM pairs in “Eighteen antagonisms and nineteen mutual inhibitions” is 2.46, which is longer than the average path distance of the network, 2.17. Because of the most commonly used TCM in formulas, liquorice root, the network path distance of the medicine pairs in “Eighteen antagonisms and nineteen mutual inhibitions” has been greatly shortened. But the difference between the special medicine pairs and the average path distance still signified that the theory of “Eighteen antagonisms and nineteen mutual inhibitions” is science based from the results of TCM formula network.

### 3.2. TCM Chemical Space

**Figure 6:**
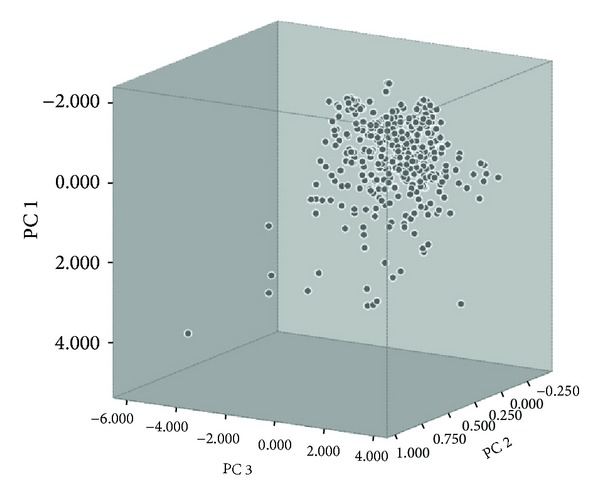
The map of TCM chemical space.

To further confirm the conclusion, we studied the chemistry basis of “antagonism and mutual restraint” in TCM by using the method of chemical space. The chemical space is constructed containing 383 commonly used TCM with 8514 chemical components. From the PCA results, we found that the variance of the first principle component (PC) was 42.72%, the second PC was 31.64%, and the third PC was 11.78%, with the cumulative rate of three PCs reaching 86.14%. Thus, it means that the three PCs included message of all descriptors basically, and the map of TCM chemical space was constructed based on these three PCs, seen in Figure 6. According to the theory of chemical space, chemical entities with similar functions tend to cluster in a certain special region. In the map of TCM chemical space, we can see that the distribution of TCM in the whole space ranges from loose to dense. But a significant proportion of them cluster at the top right corner of this chemical space. It is inferred that most of TCM are chemically compatible; in other words, they are likely to compose formulas with each other. A small proportion of TCM are dispersed and at intervals with others, which corresponds with the fact that incompatibility exists in a small proportion of TCM.

We measured the spatial distance of TCM in the chemical space, to judge the chemical affinity relationship between every two TCM. The results show that average chemical spatial distance between medicine pairs of “Eighteen antagonisms and nineteen mutual inhibitions” is 2.066, which is much larger than the average distance of all TCM, 1.641. The detailed results for each medicine pair were shown in Figure 8. This implicated that the property of incompatibility among TCM is chemically based, and it is formed by the whole chemical properties of the components contained in each TCM. 

### 3.3. TCM Metabolism Room

It was well demonstrated that the metabolic property of TCM, especially about the P450 enzymes, is always the main factor to cause the in vivo toxicity when two kinds of incompatible TCM are used together [29]. For instance, if the chemical components from two TCM in vivo compete for one special P450 enzyme, the guardian responsible for detoxifying the dangerous drugs or TCM chemical components in the body, one of them is doomed to be left with its untreated form. If this one happened to be toxic, the body will be poisoned. In this case, it will be called the incompatibility of TCM. Thus, studying the metabolic property of TCM will be helpful to reveal the incompatibility phenomenon in TCM. Accordingly, we built this TCM metabolism room, where all the TCM will be fixed in their positions by evaluating their activities towards P450 enzymes. In this room, the TCM ones close to each other indicate that they have the close metabolic characteristic. It also implies a potential risk when they are used in a prescription simultaneously. The PCA results showed that the variance of the first PC was 52.13%, the second PC was 30.54%, and the third PC was 9.69%, with the cumulative rate of three PCs reaching 92.36%. Therefore, it means that the three PCs included the most message of all descriptors, and the map of TCM chemical space was constructed based on these three PCs.

**Figure 7:**
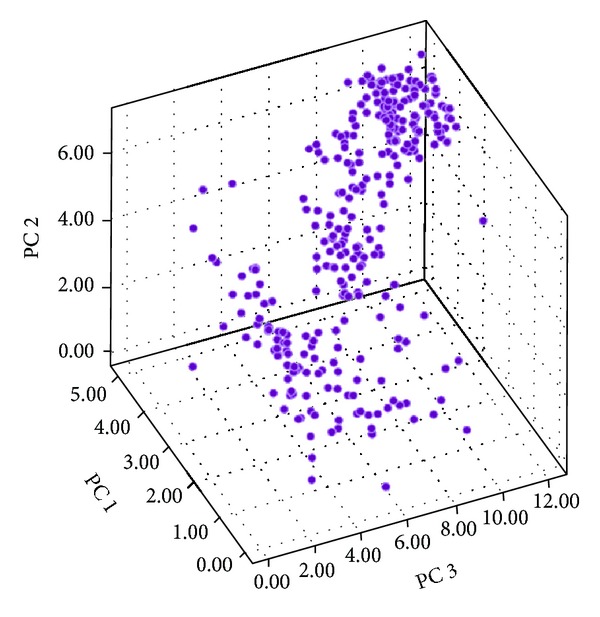
The map of TCM metabolism room.

**Figure 8:**
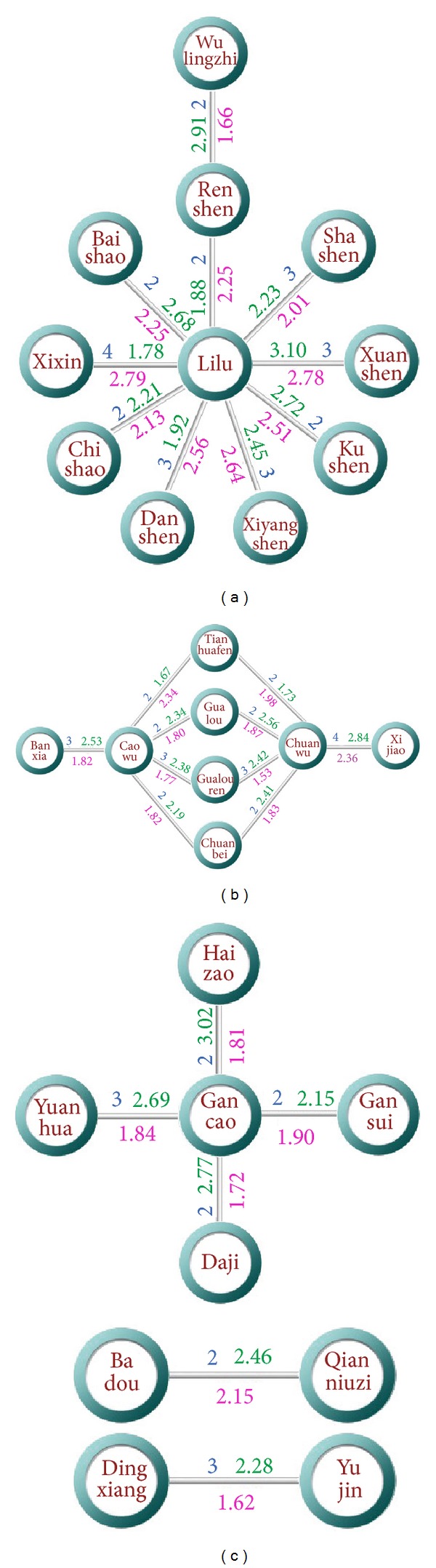
The network path distance (blue numbers), chemical spatial distance (pink numbers), and metabolism room distance (green numbers) between the medicine pairs of “Eighteen antagonisms and nineteen mutual inhibitions.”

In Figure 7, we can see that there is a large proportion of TCM located in the upper inside corner, where most of P450 enzymes keep high activity. It tells us that a large part of TCM is easily metabolized, for many P450 enzymes can undertake this job with enthusiasm. But we also can be implied that a part of TCM favor exclusive enzyme colony, with their particular position being shown in the room. The distance of TCM in the metabolism room was measured, so as to tell the metabolic relationship between every two TCM. The result shows that the average metabolism room distance between the TCM pairs in “Eighteen antagonisms and nineteen mutual inhibitions” is 2.40, which is much shorter than the average distance of all TCM, 4.25. The detailed results for each medicine pairs were shown in Figure 8. It showed that the incompatible TCM pairs tend to compete for the similar P450 enzyme colony, which make the toxic TCM ones obstructed to be metabolized or detoxified. This probably induces the incompatibility phenomenon that occurred.

### 3.4. Discussion about the Limitation of the Applied Methodology

In the construction of TCM chemical space and metabolism room, we employed a new methodology, weight center treatment. It is a novel strategy to approach describing TCM by chemical information. Chemistry is easy to be used to describe a single compound by various chemical descriptors. But TCM always contains tens or even hundreds of chemical compounds. It is a difficult problem to apply chemistry to describe a kind of TCM by batches of chemical descriptors derived from its numerous chemical components. Enlightened from geometrics, we applied weight center strategy to obtain an integrated chemical profile of a kind of TCM by treating all its chemical components in a chemical space. This chemical space is multidimension featured, every dimension of which represents a kind of chemical description. In this space, the dispersed chemical components of a kind of TCM concentrate to a pyknosis, the weight center, which will be used as the symbol of this TCM. That means a kind of TCM will be described by a series of treated chemical descriptors produced by the original descriptors of all the compounds contained in this kind of TCM. In this study, we assumed that all the “particles” in the chemical space have the same mass; that is to say, all the chemical components are assigned the same content ratios. Apparently, it was not fully considered since the content of the chemical components, in fact, is various in a kind of TCM. However, even though our software WeightCenterFinder is originally designed to be able to assign the components content values, it is impossible, in the present condition, to collect all the exact content data of the TCM chemical components. The reason is that, a part of these data are still absent; on the other hand, the available data are always in disparity because of the different measure methods and conditions. In addition, the number of the chemicals in a kind of TCM is also not completely certain, because there are still new compounds that can be discovered from TCM. But the number of the chemicals is very crucial to position the TCM weight center. These factors, obviously, will bring uncertainty and inaccuracy to the results of this study. Therefore, it is expected that, with the coming of new technologies and more discoveries, this methodology will have further improvement in the future.

## 4. Conclusion

In this study, the TCM formula network was built by using complex network methodologies. Meanwhile, we built a TCM chemical space and a TCM metabolism room under the theory of chemical space. Then the analysis of general network properties and chemical space was performed. The analysis results showed that the TCM formulas network was a partial scale-free network and also has the feature of small-world network. The analysis of TCM chemical space indicated that the properties of compatibility and incompatibility among TCM had chemical foundation. In addition, we found that the incompatible TCM pairs were different from the other ones in metabolism room. The ancient rule for incompatibility of TCM, “Eighteen Antagonisms and Nineteen Mutual Inhibitors,” was evidenced by our analysis from TCM formula network, chemical space, and metabolism room constructed in this work. Taken together, the TCM formula network, chemical space, and metabolism room gave us new insight and standpoint in analyzing the incompatibility property of TCM. We also hope it will bring up some new ideas and enlightenments into solving the problems in investigation of TCM and formulas.

## Figures and Tables

**Table 1:** The main topological parameters of TCM formulas network.

Parameter	Value
Network diameter	5
Average distance	2.17
Clustering coefficient	0.726
Average degree	31.7
